# Nurses’ experiences implementing clinical practice guidelines for detecting and managing chronic kidney disease in Eswatini’s primary healthcare facilities: A qualitative study

**DOI:** 10.4102/hsag.v31i0.3216

**Published:** 2026-07-20

**Authors:** Delisile F. Simelane, Portia J. Jordan, Wilma ten Ham-Baloyi

**Affiliations:** 1 Department of Nursing and Midwifery, Faculty of Medicine and Health Sciences, Stellenbosch University, Stellenbosch, South Africa; 2 Department of Nursing, Faculty of Health Sciences, Nelson Mandela University, Port Elizabeth, South Africa

**Keywords:** clinical practice guidelines, chronic kidney disease, early detection, primary health care, end-stage kidney disease, renal replacement therapy

## Abstract

**Background:**

Chronic kidney disease (CKD) is an emerging public health concern in Eswatini, with increasing prevalence among individuals with hypertension and diabetes. In primary healthcare facilities, professional nurses are essential to the early detection and management of CKD, guided by clinical practice guidelines and standardised protocols for non-communicable diseases.

**Aim:**

This study explored the experiences of nurses in implementing clinical practice guidelines for the detection and management of CKD in primary healthcare facilities in Eswatini.

**Setting:**

Nine selected primary healthcare facilities across Eswatini.

**Methods:**

A qualitative, descriptive, and contextual design was used. Semi-structured interviews were conducted with 25 professional nurses, using purposive sampling. Interviews were transcribed, and thematic analysis was conducted following Gibbs’ six-step framework, with ATLAS.ti used to support data management and organisation.

**Results:**

Three key themes and 12 sub-themes emerged from the data analysed. The three themes were: (1) nurses’ experience in implementing clinical practice guidelines, (2) training on the implementation process and the content of clinical practice guidelines, and (3) benefits of clinical practice guideline implementation.

**Conclusion:**

Investing in nurse training in clinical practice and its implementation is essential to improving the detection and management of CKD in Eswatini’s primary healthcare facilities.

**Contribution:**

The study provides valuable insights into the experiences of primary healthcare nurses implementing clinical practice guidelines for the early detection and management of CKD, shedding light on the challenges faced and opportunities to improve implementation in resource-constrained settings.

## Introduction

Chronic kidney disease (CKD) is a global health concern characterised by progressive and irreversible loss of kidney function (Vaidya & Aeddula 2024). It is classified into five stages based on estimated glomerular filtration rate (eGFR), with early stages (stages 1–4) often asymptomatic and stage 5 indicating end-stage kidney disease (ESKD) requiring renal replacement therapy (RRT) (Levey et al. 2020). Chronic kidney disease disproportionately affects low- and middle-income countries (LMICs) due to limited diagnostic capacity, workforce shortages, and medication stockouts (Bello et al. 2019b). Limited availability and affordability of kidney health laboratory tests, together with shortages of nephrology services, and inconsistent access to treatment, continue to hinder effective CKD management in these settings (Okpechi et al. 2021). In sub-Saharan Africa, including Eswatini, major drivers include hypertension, diabetes, HIV-associated kidney disease, infections, nephrotoxic drugs, and delayed health-seeking (George et al. 2022; Stanifer et al. 2019). These factors are compounded by the broader challenges faced in many LMICs. According to the World Health Organization (WHO), CKD is a major non-communicable disease (NCD) and a chronic health condition that progresses gradually over time and is often irreversible (WHO 2025). In Eswatini, dialysis services reflect a growing CKD burden, emphasising the importance of early detection and prevention; however, access to RRT, including dialysis and transplantation, remains limited by high treatment costs, inadequate infrastructure, and shortages of specialised healthcare personnel (Nkunu et al. 2024; Yeung et al. 2024). Strengthening early detection and management of CKD at the primary healthcare level is essential to slow disease progression, prevent complications, and reduce reliance on costly RRT (George et al. 2022; Vaidya & Aeddula 2024).

This acknowledgement highlights the essential role of nurses in applying clinical practice guidelines to deliver standardised, evidence-based care for CKD patients in primary health care (PHC) facilities (Raphael et al. 2023b). Clinical practice guidelines offer structured recommendations to guide clinical decision-making and promote patient care, particularly for NCDs such as CKD (Raphael et al. 2023a). The effective implementation of clinical practice guidelines in PHC facilities is crucial to improving CKD management by ensuring early detection, appropriate interventions, and consistent care delivery, ultimately enhancing patient outcomes in the renal population (Schwabenbauer et al. 2020).

In Eswatini’s PHC facility, nurses serve as the main providers of chronic disease care, placing them at the frontline of CKD management. They have a pivotal role in implementing clinical practice guidelines in the early detection and management of CKD. They are responsible for identifying early indicators of CKD through routine patient assessments and performing basic diagnostic tests, as well as educating patients about major risk factors such as hypertension and diabetes, as both conditions are the leading causes of CKD and can significantly accelerate kidney damage if poorly controlled. Hypertension increases pressure within the blood vessels of the kidney, gradually damaging their filtering function, while diabetes causes persistent high blood glucose levels that injure kidney tissue over time, leading to faster CKD progression, severe complications, and kidney failure requiring dialysis or kidney transplantation (Weldegiorgis et al. 2020). Additionally, they actively promote preventive strategies to reduce disease progression (Thwala, Zwane & Magagula 2023). If detected early at the PHC facility, CKD progression can be significantly slowed, leading to improved patient outcomes, reduced mortality, and decreased reliance on high-cost tertiary services such as dialysis (Mthethwa, Dlamini & Thwala 2023).

In Eswatini, PHC nurses obtain their clinical practice guidelines mainly from the Ministry of Health through National Standard Treatment Guidelines (STGs), Essential Medicines Lists (EMLs), and disease-specific guidelines and clinic-level guides for hypertension, diabetes, and kidney disease management. The application of clinical practice guidelines has been shown to improve healthcare quality by encouraging evidence-based practice, minimising inconsistencies in treatment approaches, and ensuring timely intervention for patients (Mlambo, Shabangu & Dlamini 2021). In low-resource environments like Eswatini, specialist care is often limited due to broader challenges faced by LMICs, including inadequate healthcare funding, constrained government health budgets, and competing public health priorities that reduce investment in specialised services. In this context, enabling nurses to utilise clinical practice guidelines effectively help to address critical gaps in service delivery.

This approach supports early diagnosis and management of CKD at the primary care level, which is essential for early recognition of and prevention of disease progression (Theilmann et al., 2023). Nurses facilitate prompt referrals to specialists and follow clinical practice guidelines to improve the quality of care and patient outcomes (Nozu et al. 2024). Little is known in Eswatini about PHC nurses’ experiences in implementing clinical practice guidelines for the detection and management of CKD.

The availability of clinical practice guidelines in PHC facilities across Eswatini varies considerably. While general NCD guidelines, the Package of Essential Non-communicable Disease Interventions (WHO-PEN) protocol, and the Eswatini STGs (Ministry of Health Eswatini 2022; WHO 2020) are present in many clinics, their distribution is often inconsistent (Hlophe et al. 2023b).

Furthermore, the early stages of CKD are typically asymptomatic, necessitating proactive, and protocol-driven screening and evidence-based decision-making, an approach that remains inconsistently applied across PHC facilities (Magagula & Mndzebele 2022). This gap is further compounded by insufficient training for most PHC nurses, who are often not adequately prepared to detect or manage CKD in their clinical practice (Hlophe et al. 2023a). Some nurses face challenges accessing printed copies of the guidelines, while others lack functional electronic systems to support the implementation of clinical practice guidelines (Nkambule, Mthethwa & Hlophe 2020a).

In Eswatini, nurses face high patient loads and understaffing, which limit their ability to consistently apply the basic elements of available clinical practice guidelines (Mndzebele, Khumalo & Mabuza 2022). Health system resource constraints and patient-related factors, such as geographic and economic challenges, can hamper the effective implementation of clinical practice guidelines.

There is a growing recognition of the need to adapt proven implementation frameworks, such as the Consolidated Framework for Implementation Research (CFIR), to Eswatini’s PHC context (Turner et al. 2020). Emerging evidence also emphasises the need for CKD-specific tools, structured nurse-led training programs, and digital decision support systems to facilitate timely diagnosis and management (Mthethwa et al. 2023). The study explored the experiences of PHC nurses in implementing clinical practice guidelines to identify context-specific barriers and develop practical, locally appropriate evidence-based solutions for early detection and management of CKD in Eswatini. Chronic kidney disease is a growing global public health concern marked by progressive and irreversible loss of kidney function (Vaidya & Aeddula 2024). Chronic kidney disease is classified into five stages based on eGFR. Chronic kidney disease often remains asymptomatic in its early stages (1–4), with stage 5 representing ESKD requiring RRT (Levey et al. 2020). This silent progression contributes to late diagnosis, particularly in resource-limited settings.

Chronic kidney disease disproportionately affects LMICs, where limited diagnostic capacity, workforce shortages, and medication stockouts hinder early detection and management (Bello et al. 2019a). In sub-Saharan Africa, including Eswatini, major drivers include hypertension, diabetes mellitus, HIV-associated kidney disease, recurrent infections, nephrotoxic medication use, and delayed health-seeking behaviour, resulting in advanced disease at presentation (George et al. 2022; Stanifer et al. 2019). Data services in Eswatini have revealed a growing disease burden, yet access to RRT remains limited due to high costs and infrastructure demands (Bello et al. 2019a).

Strengthening early detection and management of CKD at the PHC level is therefore essential. Preventive strategies targeting high-risk populations are more sustainable than managing advanced CKD and can slow disease progression, reduce complications, and limit dependence on costly tertiary services (George et al. 2022; Vaidya & Aeddula 2024). Nurses play a critical role in applying clinical practice guidelines to deliver standardised, evidence-based care, particularly for NCDs such as CKD (Raphael et al. 2023b; Schwabenbauer et al. 2020).

In Eswatini, nurses are the main providers of chronic disease care in PHC facilities and are central to early CKD detection, patient education, preventive care, and referral (Mthethwa et al. 2023; Thwala et al. 2023). However, inconsistent availability of clinical practice guidelines, limited training, high patient loads, and weak digital support systems constrain effective implementation (Hlophe et al. 2023a; Mndzebele et al. 2022; Nkambule et al. 2020). Emerging evidence highlights the need for CKD-specific tools, nurse-led training, and context-adapted implementation frameworks, such as the CFIR, to improve CKD care in Eswatini’s PHC context (Theilmann et al., 2023; Nozu et al. 2024; Turner et al. 2020).

### Aim of the study

This study explored the experiences of professional nurses in implementing clinical practice guidelines for the detection and management of CKD in PHC facilities in Eswatini.

## Research methods and design

### Design

A qualitative explorative, descriptive, and contextual design was used to collect data from nurses. A qualitative research design was chosen to explore and understand participants’ subjective experiences and the meanings they attach to them within real-world settings (Smith & Brown 2021). This approach is appropriate for exploring complex healthcare practices influenced by context and lived realities.

The study is exploratory due to limited research on the implementation of clinical practice guidelines for CKD in PHC in Eswatini, allowing in-depth exploration without predetermined assumptions. It is also descriptive, providing a detailed account of clinical practice guideline (CPG) implementation. Data were collected through semi-structured interviews, with verbatim quotations used to reflect participants’ views on the phenomenon.

The study is contextual, conducted within PHC facilities in Eswatini, considering local factors such as staffing shortages and resource limitations.

### Research setting

The study was conducted in Eswatini, a landlocked country in Southern Africa with a population of around 1 million (UNICEF 2022). Eswatini has four regions, namely: Hhohho, Manzini, Lubombo, and Shiselweni, and a mixed health system comprising government, mission, and private facilities. It includes 14 hospitals, 5 government health centres, 6 public health units, and 215 clinics and outreach sites.

Nine PHC facilities with high renal caseloads were purposively selected. These facilities emphasise early detection, management of risk factors such as hypertension and diabetes, and control of symptoms, given the limited availability of nephrology services.

### Population, sampling method, and sample

The population comprised 51 professional nurses who were permanently employed across the nine primary healthcare clinics in the four regions of Eswatini. Of these clinics, three were situated in Hhohho region, three in Shiselweni region, two in the Lubombo region, and one in the Manzini region. Purposive sampling was used to recruit professional nurses permanently employed in PHC facilities in Eswatini. Eligible participants had at least 6–12 months of PHC experience and were directly involved in NCD-related care, including screening, early detection, referral, or management of patients at risk. Participants were drawn from PHC facilities across all four regions of Eswatini and were willing to participate in this study. This sampling approach ensured a balanced representation of nurses across regions. It supported comprehensive data collection on nurses’ experiences with implementing clinical practice guidelines for CKD detection and management in Eswatini’s PHC facilities.

Nurses working in secondary or tertiary healthcare facilities, including hospitals and specialist renal or dialysis units, students, nursing assistants, support staff, nurses with no involvement in CKD-related care at the PHC level, and nurses employed temporarily were excluded.

To ensure equal opportunities for participation, at least three nurses from each clinic were interviewed. Twenty-five interviews were conducted across the nine facilities, after which data saturation was reached, as no new information or themes emerged.

### Data collection instrument

Data were collected through individual, face-to-face, semi-structured, in-depth interviews. A semi-structured interview guide was developed based on the literature. The guide consisted of a pre-prepared list of questions to be discussed during the interview, ensuring consistency while allowing flexibility to probe more deeply into responses when necessary. Four questions were asked: (1) Can you describe your experiences with implementing CPG for CKD within your PHC facility? (2) What role do you play in the implementation of clinical practice guidelines on CKD in the PHC context as a nurse? (3) What barriers would you consider in the implementation of clinical practice guidelines on CKD in the PHC context? (4) In your view, what strategies or changes could improve the implementation of clinical practice guidelines for CKD by nurses in PHC facilities? To establish participant profiles, demographic data were collected before each session on the scheduled interview day (Table 1).

**TABLE 1:** Participant profile.

Participant ID	Age (years)	Gender	Years of experience
P01	34	Female	10
P02	29	Male	5
P03	42	Female	18
P04	38	Male	15
P05	27	Female	4
P06	31	Female	6
P07	36	Male	12
P08	40	Female	16
P09	25	Female	3
P10	30	Male	5
P11	33	Female	8
P12	44	Female	20
P13	28	Male	4
P14	39	Female	14
P15	32	Female	9
P16	35	Male	10
P17	26	Female	2
P18	41	Male	17
P19	29	Female	6
P20	37	Male	13
P21	45	Female	21
P22	34	Male	9
P23	31	Female	7
P24	28	Female	5
P25	36	Male	11

### Data collection

After obtaining the necessary permissions from all stakeholders to conduct the study, approval for recruiting participants was obtained from the facility nurse managers, following discussions to gain access to eligible professional nurses. During a 15-min session, verbal information was provided regarding the purpose of the study, eligibility criteria, and what participation would entail. Nurses who expressed interest were given detailed verbal and written information outlining their rights as participants, the voluntary nature of participation, and the expected duration of the interviews. Written informed consent was then obtained using consent forms available in both English and siSwati, with the option to conduct the entire process in siSwati if preferred. As the researcher was fluent in both languages, translation was not required.

The interview schedule was piloted during the first interview to assess clarity, relevance, and alignment with the study objectives. As no amendments were necessary, the pilot interview was included in the main study.

Interviews were conducted by the researcher, a female with a master’s degree in nursing science, expertise in nephrology nursing and healthcare policy, and experience in qualitative research. The researcher had no prior working relationship with participants, minimising the potential for bias or power dynamics. Before each interview, verbal confirmation of written consent was obtained, and the interview process was explained in detail, allowing participants to ask questions for clarification and comfort.

Interviews were conducted in English, although participants were free to respond in their preferred language, including siSwati. Probing and paraphrasing techniques were used to elicit rich, detailed responses. Interviews took place in quiet areas within healthcare facilities or at alternative venues preferred by participants to ensure privacy and avoid disruption to service delivery. With participants’ permission, interviews were audio-recorded, and field notes were taken to capture observations and contextual details. Interviews lasted for 40 min – 60 min and were conducted between July and August 2024.

### Data analysis

Each interview required approximately 4–6 h to transcribe. Thematic analysis was conducted following Gibbs’ six-step framework (Gibbs 2007). This systematic and iterative process ensured rigour and transparency in analysing qualitative data. The researcher followed a systematic process of data analysis. Firstly, she familiarised herself with the data by repeatedly reading the interview transcripts and field notes to deepen her understanding of the content. Initial coding was then conducted, during which relevant sections of the text were identified and assigned codes. Similar codes were subsequently grouped to form preliminary categories and emerging themes.

Secondly, these themes were reviewed and refined to ensure that they accurately reflected the data and aligned with the study objectives. Thereafter, the themes were clearly defined and appropriately named to capture their underlying meanings. Finally, the findings were presented in narrative form and supported by verbatim quotations from participants to enhance credibility and strengthen interpretation. ATLAS.ti (Berlin, Germany), a qualitative data analysis software, was used to support data management and organisation (Khokhar et al. 2020). Transcripts were read repeatedly to achieve familiarisation before being imported into ATLAS.ti for initial open coding. Codes were iteratively compared and grouped into categories, from which three overarching themes and 12 sub-themes were developed. An independent co-coder reviewed a subset of transcripts and coding decisions, and discrepancies were resolved through discussion, enhancing the credibility and dependability of the analysis.

### Trustworthiness

Trustworthiness was ensured through strategies by Lincoln and Guba (1985), addressing credibility, dependability, confirmability, and transferability. Credibility was enhanced through member checking, prolonged engagement within the primary healthcare context, peer debriefing with academic supervisors, and negative case analysis to refine emerging interpretations. Dependability was supported by maintaining a clear audit trail documenting methodological and analytical decisions. Confirmability was strengthened through reflexive journaling and the use of verbatim participant quotations, ensuring that the findings were grounded in the data. Transferability was facilitated through detailed descriptions of the study context and participants, enabling readers to assess the applicability to similar settings.

### Ethical considerations

Ethical approval for the study was obtained from the Stellenbosch University Health Research Ethics Committee HREC Reference No: S23/07/163 (PhD) and the Eswatini Ministry of Health Research Ethics Committee (Protocol No: EHHRRB 009/2024). Permission to conduct the study was also granted by the Ministry of Health and the PHC nurse managers before data collection. The study adhered to ethical principles guided by the Belmont Report (2016), including privacy, confidentiality, beneficence and non-beneficence, and anonymity, as pseudonyms were used to maintain participant confidentiality, and data access was limited to the researcher. Written consent was obtained from participants before the interviews.

## Results

Table 1 presents demographic data and professional background information of the 25 study participants, including their age, gender, and years of experience. Of the 25 participants, 14 were female, and 11 were male, aged 25–45 years (mean age 33.9 years), with 2–21 years (average 10 years) of experience. All 25 participants were full-time employed professional nurses at the PHC facilities.

The analysis identified three themes and 12 sub-themes related to the experiences of professional nurses in implementing clinical practice guidelines for CKD in the PHC facilities, as shown in Table 2.

**TABLE 2:** Themes and sub-themes.

Theme	Sub-themes	Categories
1. Nurses’ experience in implementing clinical practice guidelines	1.1. Nurse-related factors	Individual nurse competency, attitudes, and beliefs
	1.2. Lack of resources	Insufficient staffing, equipment, and materials
	1.3. Time constraints	Workload pressures limiting guideline application
	1.4. Accessibility to clinical practice guidelines	Availability and ease of accessing guideline documents
	1.5. Resistance to change	Reluctance to adopt new practices or protocols
2. Training on the implementation process and content of clinical practice guidelines	2.1. Guideline implementation	Strategies and processes for introducing clinical practice guidelines
	2.2. Adherence to available guidelines	Compliance and consistency in following guidelines
	2.3. Training for a leader or mentor for clinical practice guideline implementation	Leadership development for guiding clinical practice guideline adoption
3. The benefits of clinical practice guideline implementation	3.1. Standardisation of care protocols through guidelines	Uniform practices ensuring consistent patient care
	3.2. Better care leads to healthier patients and fewer complications	Improved patient outcomes and reduced adverse events
	3.3. Enhanced nurse roles to implement patient education and comprehensive care	Expanded nursing responsibilities in patient-centred care
	3.4. Integration of technology	Use of digital tools to support clinical practice guideline implementation

### Theme 1: Nurses’ experience in implementing clinical practice guidelines

Nurses’ experience in implementing clinical practice guidelines reflects both benefits and challenges in clinical practice. Clinical practice guidelines are valuable for improving the quality, consistency, and accuracy of care, supporting early detection, decision-making, and patient education. However, their implementation is often limited by nurse-related factors, lack of resources, time constraints, accessibility to clinical practice guidelines, and resistance to change.

#### Sub-theme 1.1: Nurse-related factors

Participants described their experiences implementing clinical practice guidelines as shaped by challenges in nursing practice. They shared that high patient volumes and significant time constraints within the PHC setting limited their ability to fully implement the guidelines. The heavy workload created considerable pressure, making it difficult for nurses to consistently deliver quality, evidence-based, and guideline-directed care within the available time:

‘The CKD clinical guidelines are available, though not very detailed, but it’s difficult to implement them properly because of the high patient volume. Many patients rely on hospital transport and are often in a rush, which limits the time we have for thorough assessments and proper engagement.’ (P17, 26-year-old, female)

‘We always aim to follow the CKD guidelines step by step, but when patients are anxious about missing their transport and ask us to speed things up, we’re forced to adjust or skip certain parts of the process just to accommodate their time constraints.’ (P12, 44-year-old, female)

#### Sub-theme 1.2: Lack of resources

Participants highlighted that the shortage of essential resources, such as human, financial, and infrastructural resources, significantly undermines the effective implementation of clinical practice guidelines for CKD in PHC facilities.

Inadequate staffing limits the time available for thorough patient assessments and adherence to CPG-based care.

Insufficient funding restricts access to training, educational materials, and essential supplies needed to operationalise the guidelines. Moreover, poor infrastructure, such as unreliable electricity, limited space, and a lack of digital tools, further hampers the integration of clinical practice guidelines into routine practice:

‘We’re too short-staffed to follow the CKD guidelines properly; there just isn’t enough time to assess each patient thoroughly when you’re seeing over a hundred people a day. Without enough hands, it’s hard to apply the guidelines the way they’re meant to be used.’ (P19, 29-year-old, female)

‘Even if we have the CKD guidelines, using them effectively is a struggle. There’s no funding for training or materials, and with issues like unreliable power, cramped consultation rooms, and no access to digital tools, it becomes almost impossible to fully implement the guidelines in our daily work.’ (P8, 40-year-old, female)

#### Sub-theme 1.3: Time constraints

Participants reported that time constraints hinder the effective use of CKD management guidelines. They found the guidelines lengthy and difficult to consult during busy hours, with CKD care, especially when managing comorbidities, being time-consuming. Short consultation times and competing priorities further limited their ability to fully adhere to the guidelines in daily practice:

‘Time constraints are a significant issue. With our busy schedules and the complexity of CKD management, it’s often difficult to fully adhere to all aspects of the guidelines during short consultation times.’ (P14, 39-year-old, female)

‘The CKD guidelines are helpful, but they don’t offer much beyond what’s already in the standard NCD treatment guidelines. They come in book format, which makes it difficult to read during our busy clinic hours. Managing CKD, especially when patients have other conditions, takes time, and with short consultations and high patient numbers, it’s challenging to follow every step as recommended.’ (P11, 33-year-old, female)

#### Sub-theme 1.4: Accessibility to clinical practice guidelines

Participants conveyed that limited access to CKD guidelines and a lack of training hinder their effective use. There are insufficient printed copies, no budget for workshops or refresher sessions, and many nurses are unfamiliar with the guidelines, often sharing the few available copies:

‘We have very limited access to the CKD guidelines; there aren’t enough printed copies for everyone, so we have to take turns using the few that are available. Most of us haven’t received any formal training on how to use them properly, mainly because there’s no budget for workshops or refresher courses.’ (P3, 42-year-old, female)

‘The lack of funding affects our ability to use the CKD guidelines effectively. There’s not enough money to print more copies or to organise training sessions, and as a result, many nurses are unfamiliar with the content and how to apply it in practice.’ (P25, 36-year-old, male)

#### Sub-theme 1.5: Resistance to change

Participants noted that some experienced nurses are resistant to adopting the new practices introduced by the CKD guidelines, as they tend to continue doing what they have always done. This makes it harder to implement the changes because younger staff often follow their lead. Further, participants expressed that the reasons for the resistance might be insufficient training on the guidelines and concern that the new protocols will add more work to their already heavy load:

‘One of the biggest challenges we face is resistance from experienced staff who are used to doing things a certain way. They’re often hesitant to change their routines, even when new guidelines are introduced, which slows down implementation efforts.’ (P7, 36-year-old, male)

‘Sometimes nurses are quick to dismiss updated information from workshops or training sessions. If they feel the changes will increase their workload or disrupt familiar practices, they tend not to take the new guidelines seriously.’ (P4, 38-year-old, male)

### Theme 2: Training on the implementation process and the content of clinical practice guidelines

Training on the implementation process and content of clinical practice guidelines is essential for supporting nurses in effectively applying guidelines in clinical practice. Nurses emphasise the importance of continuous education and capacity building to enhance their understanding of guideline content, improve competence, and ensure proper implementation. Adequate training also promotes confidence, consistency in care delivery, and better patient outcomes. The participants recognised the importance of guideline implementation and adherence, as well as the need for training leaders or mentors for clinical practice guideline implementation.

#### Sub-theme 2.1: Guideline implementation

Participants recognised the critical role of clinical practice guidelines in guiding their clinical decision-making, standardising care, and improving patient outcomes, especially in resource-limited environments. Implementation involved more than simply making the guidelines available; it required deliberate integration into daily practice, which often included adapting the guidelines to local contexts, training staff, and creating user-friendly tools to support adherence. Participants described how incorporating clinical practice guidelines into their workflow enabled them to deliver more accurate, consistent, and evidence-based care:

‘Implementing the guidelines has become part of our routine; we use them to assess, manage, and refer patients. They give us structure, especially when dealing with chronic conditions like CKD. Even with limited resources, we do our best to follow them closely.’ (P9, 25-year-old, female)

‘We didn’t just receive the guidelines; we made sure they were usable. We trained our team, simplified some parts into quick-reference charts, and ensured everyone understood how to apply them in our context. That’s how we’ve been able to make them work here.’ (P5, 27-year-old, female)

Despite highlighting the importance of clinical practice guidelines, participants identified a notable gap in ongoing professional development related to clinical practice guidelines and CKD management. They highlighted inadequate and inconsistent training opportunities, with some mentioning missed or ineffective workshops. This lack of continuous education contributes to low confidence, resistance to adopting new guidelines, and reluctance stemming from prior negative experiences:

‘There is a gap in ongoing professional development focused specifically on clinical practice guidelines and CKD management.’ (P24, 28-year-old, female)

‘Sometimes we are invited to attend a workshop, but we eventually lose that workshop.’ (P20, 37-year-old, male)

‘Inadequate training contributes to resistance to new guidelines due to a lack of confidence and previous negative experiences. If healthcare providers feel ill-equipped… they may resist adopting them altogether.’ (P6, 31-year-old, female)

#### Sub-theme 2.2: Adherence to available guidelines

The participants consistently described their commitment to and adherence to clinical practice guidelines by conducting comprehensive patient assessments, including detailed history-taking, physical examinations, and monitoring vital signs. Based on these evaluations and available resources, they make informed diagnoses and ensure timely referrals to higher-level care when necessary:

‘I make sure to follow the guidelines correctly by conducting thorough assessments, taking patient history, checking vitals, and performing physical exams, so I can make accurate diagnoses and manage care appropriately. If advanced care is needed, I refer the patient without delay.’ (P23, 31-year-old, female)

‘We do everything from assessment to diagnosis and management based on the available clinical practice guidelines, but when we lack the necessary equipment, we refer patients to higher-level facilities.’ (P1, 34-year-old, female)

#### Sub-theme 2.3: Adherence to available guidelines Training for a leader or mentor for clinical practice guideline implementation

Participants highlighted the value of taking an active leadership role in adapting CKD guidelines to the specific needs of PHC facilities and in providing ongoing training through in-service education. They emphasised the importance of mentorship, in which experienced nurses guide and support newly employed staff and students. In resource-constrained settings, pairing less experienced personnel with seasoned nurses was seen as an effective way to build capacity, improve understanding of the guidelines, and promote better adherence in day-to-day practice:

‘We’ve had to adapt many of the guidelines to suit our setting, and I’ve been leading the effort to train our staff. I also mentor students and newly employed nurses to ensure they understand the guidelines and can apply them effectively in practice.’ (P13, 28-year-old, male)

‘Mentorship was seen as essential, especially pairing experienced nurses with new staff who bring tech skills. This kind of support fosters better adherence to the guidelines and builds overall capacity in our resource-limited setting.’ (P21, 45-year-old, female)

### Theme 3: The benefits of clinical practice guideline implementation

Clinical practice guidelines improve healthcare quality by promoting standardised, evidence-based care. They support better clinical decision-making, enable early detection and effective management of conditions such as CKD, and improve patient outcomes. Clinical practice guidelines also streamline workflows, reduce practice variation, and enhance healthcare providers’ confidence and competence, contributing to more efficient patient-centred care.

The participants identified that clinical practice guidelines ensure the standardisation of care protocols and lead to better care, healthier patients, and fewer complications. Furthermore, clinical practice guideline implementation enhances the role of nurses in patient education and comprehensive care delivery and facilitates the integration of technology.

#### Sub-theme 3.1: Standardisation of care protocols through guidelines

Participants noted that clinical practice guidelines provide a clear and structured framework for screening and managing various health conditions, making their work more accurate and manageable. By standardising practices across healthcare providers, clinical practice guidelines ensure that all patients, especially those at high risk, receive evidence-based, reliable care. This uniformity not only improves disease monitoring but also contributes to better patient outcomes, as practitioners are aligned in their approach to treatment and prevention:

‘As a nurse, I’m guided by the clinical practice guidelines; reading and referring to them has made our work more accurate and manageable. They offer a clear framework for screening and managing conditions, which means our patients, especially those at high risk, are receiving more consistent and reliable care.’ (P18, 41-year-old, male)

‘The clinical practice guidelines have significantly improved how we approach patient care by standardising screening and management practices. In our clinic, they ensure every practitioner follows the same evidence-based protocols, which have not only improved disease monitoring but also led to better outcomes for high-risk groups.’ (P22, 34-year-old, male)

#### Sub-theme 3.2: Standardisation of care protocols through guidelines Better care leads to healthier patients and fewer complications

Clinical practice guidelines play a role in improving patient outcomes through better care in terms of early detection, timely intervention, and consistent care. Nurses report a noticeable reduction in the progression of chronic conditions, such as end-stage renal disease, attributing this improvement to the proactive use of clinical practice guidelines for earlier diagnosis and stricter management. The guidelines help ensure that patients receive appropriate tests and interventions at the right time, minimising the risk of both over-treatment and missed warning signs. Additionally, clinical practice guidelines support timely referrals when necessary, further enhancing patient care:

‘Over the past 3 years, we’ve seen a reduction in patients progressing to end-stage renal disease, and it’s largely because of earlier detection and stricter management based on the guidelines; we’re catching it before it gets worse.’ (P19, 29-year-old, male)

‘Using the clinical practice guidelines has made our care more consistent and effective. Patients get the right tests at the right time; we avoid over-treating or missing signs; and, when needed, we refer early to specialists. It’s rewarding to see how these small changes add up to better patient outcomes.’ (P1, 34-year-old, female)

#### Sub-theme 3.3: Standardisation of care protocols through guidelines Enhanced nurse roles to implement patient education and comprehensive care

Nurses highlighted the vital role they play as frontline providers in PHC and how clinical practice guidelines support their routine practice. Nurses report that while there is no specific guideline for CKD, they effectively rely on existing NCD clinical practice guidelines to guide patient screening, medication management, and lifestyle education. These guidelines have become part of their everyday workflow, helping them respond confidently and consistently to patient needs. In Eswatini, the current practice is that although the national guidelines provide limited specific information on CKD management, nurses do not wait for a dedicated CKD protocol before taking action. This highlights the practicality and accessibility of NCD clinical practice guidelines in strengthening comprehensive, evidence-based care delivery within limited resource settings such as Eswatini:

‘As nurses, we are often the first point of contact for patients in primary care. The guidelines for conditions like hypertension and diabetes have become part of our routine; they help us know exactly what to do. Even though we don’t have a CKD guideline, we still use what’s available to make sure patients at risk are screened and advised properly.’ (P16, 35-year-old, male)

‘We rely heavily on the NCD guidelines because they’re practical and always within reach. For CKD, we may not have a dedicated protocol, but we integrate recommendations from diabetes and hypertension guidelines to monitor kidney function, provide lifestyle education, and manage medications. to our patients who were informed through clear communication in their preferred language.’ (P2, 29-year-old, male)

#### Sub-theme 3.4: Integration of technology

To address the challenges of limited accessibility and to ensure consistent application of clinical practice guidelines in underserved and remote settings, several participants reported adopting digital innovations and mobile health interventions. These technologies not only helped bridge the gap between patients and healthcare services but also facilitated more standardised, guideline-informed care delivery across contexts:

‘Since starting the mobile outreach, our follow-up rates have improved significantly. Patients are more likely to continue treatment and attend review appointments, which helps us apply the guidelines more thoroughly and consistently.’ (P15, 32-year-old, female)

‘We’ve started using telemedicine and remote monitoring systems to stay connected with patients after they leave the clinic. It allows us to do medication reviews, manage symptoms, and detect issues early, following the guidelines closely, even from a distance.’ (P10, 30-year-old, male)

## Discussion

This qualitative study explored the experiences of nurses in implementing clinical practice guidelines for CKD detection and management in PHC facilities in Eswatini. The analysis of participant narratives generated three main themes and 12 sub-themes, reflecting the multifaceted experiences of nurses in implementing clinical practice guidelines for CKD in PHC facilities.

The results of this study highlighted nurses’ experience in implementing clinical practice guidelines in PHC facilities. Nurses working in these environments reported that, while clinical practice guidelines offer valuable frameworks for guiding care, several barriers hinder their full integration into everyday practice. Factors such as staff shortages, heavy workloads, inadequate resources, and limited training reduce adherence to guideline recommendations. Strengthening resource availability, ongoing professional training, and institutional support is necessary to improve compliance with clinical practice guidelines. These findings are consistent with current literature and underscore the systemic, structural, and human resource challenges present in low-resource healthcare environments (Olowu et al. 2020).

Participants frequently reported time constraints as a major barrier to clinical practice guideline implementation, citing overcrowding and limited consultation durations. These challenges are representative of time constraints, which limit nurses’ ability to apply comprehensive guidelines implementation during brief consultations (Munyewende, Rispel & Chirwa 2020). Furthermore, a lack of resources was evident, with participants highlighting staff shortages, inadequate training opportunities, and limited access to necessary technologies. This aligns with findings by Grimmer et al. (2019), who argue that without sufficient human and infrastructural resources, the uptake and consistent application of guidelines remain suboptimal. Together, these findings reinforce the factors that influence clinical practice guideline implementation, particularly in under-resourced primary healthcare settings.

This difficulty is further exacerbated by limited access to the guidelines themselves. Many nurses reported having few or no physical copies, especially CKD-specific materials. These concerns are echoed in global research, which indicates that dissemination of disease-specific guidelines is often poor in rural and low-income settings (Chansa, Chitah & Kachimba 2022). Evidence-based practices are consistent with findings from Raphael et al. (2023c), who describe how institutional inertia and fear of change can obstruct progress in clinical settings. In their study, they found that healthcare professionals often resist new guidelines due to established routines, perceived increases in workload, and uncertainty about the outcomes of implementation. This reluctance is not solely individual but often stems from broader organisational cultures that favour the status quo over innovation.

Greenhalgh et al. (2022) highlighted that even when evidence-based interventions are well-supported and accessible, uptake remains low in facilities where change is perceived as risky or disruptive. This is further echoed by Whitehead, Taket and Smith (2021), who found that healthcare workers often feel overwhelmed by the constant evolution of clinical guidelines, especially when adequate training and leadership support are lacking. They emphasised that without a supportive infrastructure, efforts to implement evidence-based practice may encounter passive or active resistance.

Training emerged as a vital but under-resourced component of successful clinical practice guideline implementation. Participants highlighted the need for structured training on guideline content and practical application. Abimbola et al. (2020) found that consistent education, mentorship, and leadership are crucial for health workers to adopt new guidelines. Those who had received mentorship or facilitated training felt empowered, suggesting that leadership development and peer mentorship are effective strategies. This finding highlights that mentorship and task-shifting are effective strategies for enhancing nurses’ ability to implement clinical practice guidelines for CKD in primary healthcare, including in Eswatini. The need for training and mentorship among participants aligns with the WHO’s (2021) recommendation to enhance guideline uptake in PHC through mentorship and task redistribution. This approach is particularly vital in the study setting, given the relatively young age and diverse clinical experience of the participants, ensuring they receive the support necessary to confidently apply clinical guidelines in practice.

Implementing clinical practice guidelines in PHC by nurses offers several important benefits; it helps standardise care, ensuring that all patients receive evidence-based, high-quality treatment regardless of where they are seen. This consistency improves patient safety and reduces variations in practice (Kitson et al. 2019). Clinical practice guidelines support early detection and better management of chronic diseases, such as diabetes and hypertension, and CKD, which are common in PHC facilities. By following clear clinical practice guidelines, nurses can intervene sooner, preventing complications and improving long-term health outcomes (Marais, Ntshoe & Moolla 2023). Using clinical practice guidelines strengthens nurses’ confidence and clinical decision-making skills. Having clear guidance empowers them to act decisively, reduces uncertainty, and enhances professional development (Camargo et al. 2022). CPG implementation promotes more efficient use of resources. It helps avoid unnecessary tests or treatments, improving cost-effectiveness and ensuring that limited supplies are used appropriately (Brouwers et al. 2019). The implementation of clinical practice guidelines often facilitates the integration of technology, such as electronic health records, clinical decision support systems, and mobile health tools, which further standardise care and enhance transparency (Sutton et al. 2020). Several studies have shown that integrating technology alongside clinical practice guidelines improves adherence, supports real-time guideline updates, and strengthens patient-provider communication, all of which contribute to building trust and improving outcomes (Khalifa 2014).

Adherence to clinical practice guidelines fosters patient trust. Patients are more likely to follow care plans and remain engaged when they see that care is systematic and based on the latest evidence, ultimately leading to better patient satisfaction and stronger nurse–patient relationships (Stacey et al. 2021).

### Study limitations

During this study, the researcher acknowledged the possibility of bias during data collection, analysis, and interpretation. To minimise its influence and ensure trustworthiness, bracketing (epoché) was applied by identifying and setting aside personal assumptions and preconceptions about the implementation of clinical practice guidelines for CKD in PHC facilities. Reflexive journaling documented decisions and potential influences throughout the study. Systematic thematic analysis and consistent use of a standardised interview guide ensured that all participants were asked similar questions, enhancing rigour and credibility. Participants’ awareness of being part of a study may also have affected their responses, with some nurses potentially portraying their adherence to clinical practice guidelines more positively than in routine practice. In addition, reliance on self-reported data introduces the possibility of recall and information bias, as variations in memory and understanding of CKD guidelines may have affected the accuracy and depth of the information provided. Together, these factors may have limited how fully the findings reflect everyday implementation challenges within Eswatini’s primary healthcare settings.

As an LMIC with limited resources, Eswatini’s healthcare setting likely influenced the depth and generalisability of the findings. To overcome the contextual limitations identified in this study, future research should focus on conducting multi-site studies across diverse PHC facilities in Eswatini, including rural, peri-urban, and urban areas. Comparative studies involving other LMICs could also help determine which findings are context-specific and which are more broadly applicable.

## Conclusion

Nurses in Eswatini’s PHC settings play a central role in implementing clinical practice guidelines for the early detection and management of CKD. Their experiences reflect the strengths and challenges of translating evidence-based recommendations into routine practice. Many nurses indicate that clinical practice guidelines provide structure, clarity, and improved consistency in the screening and management of CKD and other chronic diseases.

However, implementation is not without its challenges, including limited resources, high patient loads, staff shortages, and time constraints. Chronic kidney disease guidelines at the PHC level are often brief, lack sufficient detail, and are not easily accessible, making them challenging to apply in complex cases. To overcome these issues, it is recommended to develop comprehensive, context-specific guidelines designed specifically for PHC facilities, complemented by practical tools such as simplified algorithms and decision aids to better support nurses. Ensuring these guidelines are readily available in both print and digital formats can further promote their consistent use in everyday practice.
